# Left ventricular strain echocardiography in advanced uremic cardiomyopathy compared to dilated cardiomyopathy

**DOI:** 10.1186/s43044-023-00393-y

**Published:** 2023-07-26

**Authors:** Rizan Mohammadi, Farnoosh Larti, Roya Sattarzadeh Badkoubeh, Maryam Mehrpooya, Akram Sardari

**Affiliations:** Department of Cardiology, Imam Khomeini Hospital Complex, Tehran University of Medical Sciences, Keshavarz Boulevard, Tehran, 1419733141 Iran

**Keywords:** Dilated cardiomyopathy, Speckle tracking echocardiography, End stage renal disease, Global longitudinal strain, Segmental longitudinal strain

## Abstract

**Background:**

Cardiac involvement is common in end-stage renal disease patients. The presenting study aimed to evaluate the global and segmental longitudinal strain in patients with advanced uremic cardiomyopathy (AUCM) and compare it to dilated cardiomyopathy (DCM).

**Results:**

The mean global longitudinal strain (GLS) was significantly lower in AUCM (*P* value = 0.045). Comparing segmental strain showed a lower strain in mid inferoseptal (*P* value = 0.048), base and mid anterolateral (*P* value = 0.026, 0.001 respectively), base and mid anteroseptal (*P* value = 0.005, 0.009 respectively), base and mid inferior (*P* value = 0.015, 0.034 respectively) and mid anterior (*P* value = 0.015) in patients with AUCM compared with DCM. In both groups, the segmental strain increased from base to apex.

**Conclusions:**

Segmental and GLSs in advanced uremic cardiomyopathy were significantly lower than those of dilated cardiomyopathy. In both groups, the segmental strain increased from base to apex.

## Background

Cardiovascular complications are the important causes of mortality and morbidity in patients with chronic kidney disease (CKD) and end-stage renal disease (ESRD) [1]. “Uremic cardiomyopathy” generally expresses cardiac involvement in patients with CKD and ESRD. Various imaging and epidemiological studies have shown that the most crucial pathological change associated with uremic cardiomyopathy (UCM) is left ventricular hypertrophy (LVH). LVH is considered the primary landmark of UCM. Increased left ventricular (LV) mass, LV dilatation, and systolic and diastolic dysfunction constitute other echocardiographic abnormalities of UCM [2]. In adults, left ventricular hypertrophy and systolic dysfunction are evident even in the early stages of renal dysfunction. Cardiac abnormalities in end-stage renal disease (ESRD) have complex etiologies, including hypertension, volume overload, anemia, mineral abnormalities, coronary artery disease, and uremic toxins [3].

Speckle-tracking echocardiography (STE) allows the evaluation of myocardial deformation and heart mechanics [4, 5]. In patients with chronic renal failure, the progression of renal dysfunction (assessed by glomerular filtration rate measurement) is accompanied by a significant decrease in cardiac strain values [6, 7]. In patients with ESRD, a slight reduction in longitudinal strain may be an important marker of increased mortality [8].

Most of the presenting studies with STE focused on ESRD patients with normal left ventricular function. In this study, we focused on the advanced stages of UCM. The results of the presenting study compared the global and segmental longitudinal strain in advanced uremic cardiomyopathy with dilated cardiomyopathy.

## Methods

### Study type and population

This single-center comparative observational study was performed between March 2017 and March 2019. Written informed consent was obtained from study participants. The ethics committee approved the study protocol. Twenty-five patients aged 18 to 70 years suffering from ESRD and advanced uremic cardiomyopathy (AUCM) were included. All the ESRD patients were on routine hemodialysis via arteriovenous fistula (AVF) and were candidates for kidney transplantation. The result of the invasive coronary angiography in ESRD patients was normal; an AVF-associated high-output cardiac failure was ruled out with echocardiography and ultrasonographic flow assessment of the fistula. Advanced uremic cardiomyopathy was arbitrarily defined as a left ventricular ejection fraction (LVEF) of less than 40%. Twenty-five known patients with dilated cardiomyopathy (DCM) matched with the same left ventricular ejection fraction ranges were enrolled as the control group. All the DCM patients had normal invasive coronary angiography results in their records.

### Exclusion criteria

In patients with AUCM, several exclusion criteria were defined, including a history of heart failure or documented LVEF less than 50% before initiating renal replacement therapy (RRT), uncontrolled hypertension, congenital heart disease, pericardial disease, and history of autoimmune disorders.

In both AUCM and DCM groups, a history of diabetes mellitus, permanent pacemaker, left bundle branch block, atrial fibrillation rhythm, and inadequate acoustic window was considered exclusion criteria.

### Echocardiography

**Fig. 1:**
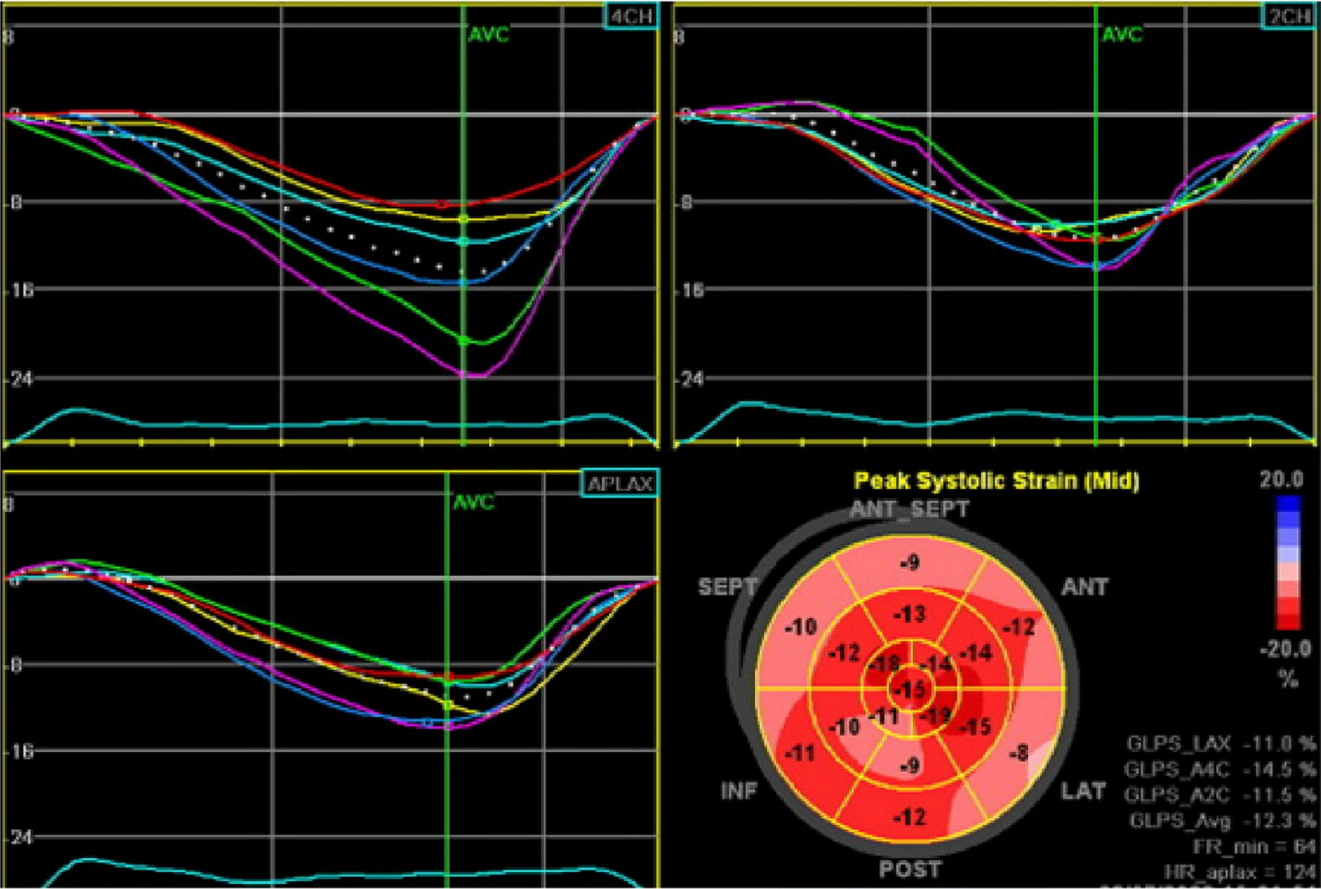
Diagram of global and segmental longitudinal strains in 17 LV myocardial segments

Transthoracic echocardiography was performed by Vivid E9 echo vendor (GE company). In ESRD patients, echocardiographic interrogation was done 6–24 h after the last hemodialysis session. All the ESRD patients had controlled blood pressure(systolic blood pressure less than 140 mmHg) at the time of echocardiography. A Cardiologist with a fellowship in echocardiography performed two-dimensional, color Doppler echocardiography, tissue Doppler imaging (TDI), and 2D STE. The left ventricular dimensions were measured by 2D echocardiography. The modified Simpson's method assessed left ventricular volumes and systolic function. Right ventricular systolic function was evaluated by tricuspid annular plane systolic excursion (TAPSE), right ventricular fractional area change (RVFAC), and right ventricular peak systolic myocardial velocity (RVSm). Valvular heart disease severity was assessed based on the latest ASE guidelines [9]. Pulmonary artery pressure (PAP) was measured by tricuspid regurgitation (TR) velocity and modified Bernoulli formula. LV diastolic function was assessed by the Doppler study of mitral valve inflow and TDI study of the septal and lateral mitral annulus [10]. Global longitudinal strain (GLS) and segmental longitudinal strain in 17 left ventricular segments were measured by 2D STE (Fig. 1). The left ventricular mass was assessed by the Devereux method [11].

### Endpoints

Global longitudinal strain, segmental longitudinal strain, and all echocardiographic measurements were compared between the two groups as the study endpoints.

### Statistical analysis

Descriptive analysis was used to describe the data, including mean ± standard deviation (SD) for quantitative variables and frequency (percentage) for categorical variables. The Chi-square, t-test, or Mann–Whitney U tests were used to compare variables. The statistical software IBM SPSS Statistics for Windows version 22.0 (IBM Corp. Released 2013, Armonk, New York) was used for the statistical analysis. P values < 0.05 were considered statistically significant.

## Results

### Baseline and demographic data

This study enrolled 25 patients with ESRD and advanced uremic cardiomyopathy (AUCM as the case group) and 25 DCM patients (as the control group). Regarding demographic characteristics, the male gender was 76.0% in AUCM and 72.0% in the DCM group (*P* = 0.877). The mean age of the AUCM group was lower than DCM (40.12 ± 12.86, vs. 50.88 ± 12.97 years, *P* = 0.005). Compelling cardiovascular risk profile, 23 patients in advanced uremic cardiomyopathy had a history of hypertension versus one in the DCM group (*P* value = 0.001). No difference was found between the two groups in the history of smoking and hyperlipidemia (Table 1). In the advanced uremic cardiomyopathy group, the mean duration of renal replacement therapy via AVF was 2.63 ± 1.34 years (range 1.5–6 years).

**Table 1:** Demographic and clinal characteristics of dilated cardiomyopathy (DCM) and advanced uremic cardiomyopathy (AUCM) patients

Values	DCM^a^ group	AUCM^b^ group	*P* value
Male gender, *n* (%)	18 (72.0%)	19 (76.0%)	0.877
Mean age (year) ± SD	50.88 ± 12.97	40.12 ± 12.86	0.005
Hypertension	1 (4.0)	23 (92.0)	0.001
Hyperlipidemia	0 (0.0)	1 (4.0)	0.998
Smoking	0 (0.0)	3 (12.0)	0.092
Mean BSA^c^ (m^2^) ± SD	1.89 ± 0.17	1.71 ± 0.17	0.001
Duration of hemodialysis (years) ± SD	N/A^d^	2.63 ± 1.34	N/A
Sessions of hemodialysis Per week (days)	N/A	3	N/A

^a^Dilated Cardiomyopathy

^b^Advanced Uremic Cardiomyopathy

^c^Body Surface Area

^d^Not applicable

### Echocardiographic data

**Fig. 2:**
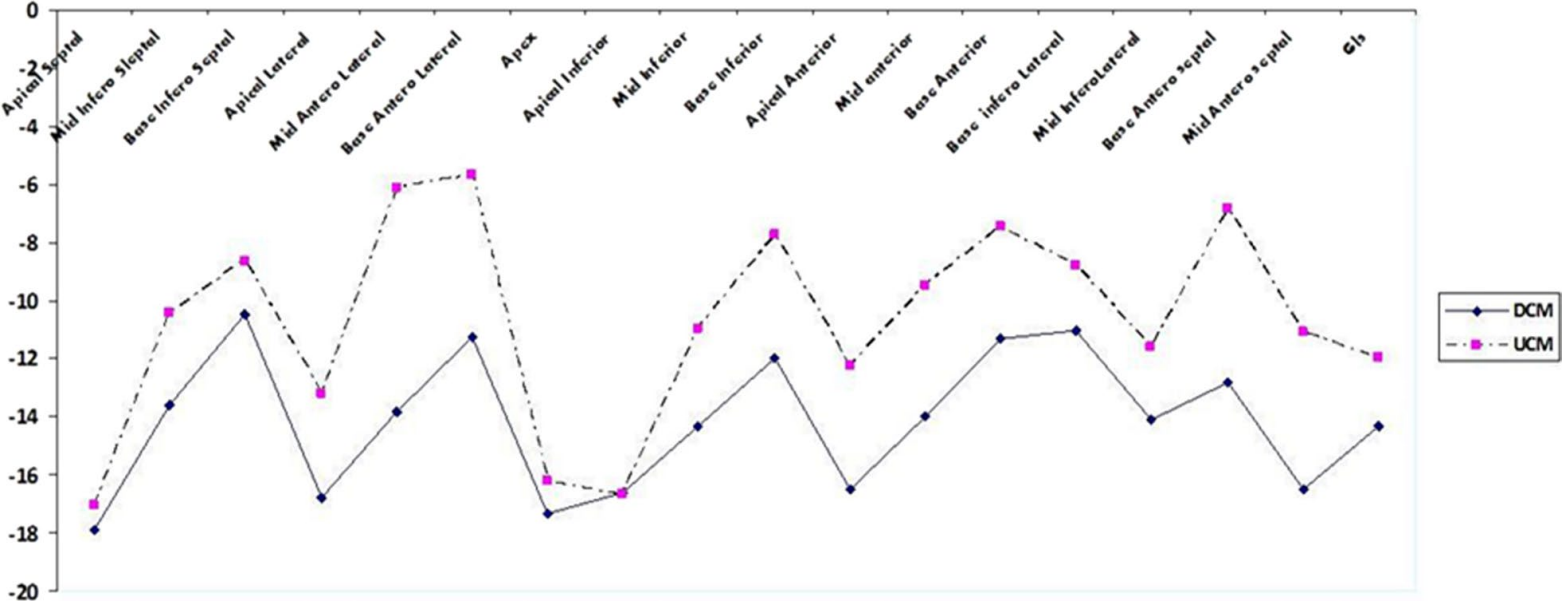
Comparison of segmental longitudinal strains of the left ventricle LV in two groups with advanced uremic and dilated cardiomyopathy

Echocardiographic data were compared in Table 2. In advanced uremic cardiomyopathy, the mean GLS was significantly lower than the DCM group (− 11.98% ± 4.88% in AUCM vs. − 14.34% ± 4.81% in DCM, *P* = 0.045). A comparison of segmental longitudinal strain in 17 segments was provided (Table 3, Fig. 2). Assessment of longitudinal segmental strains showed a significant difference in these segments: mid inferoseptal (*P* = 0.048), base anterolateral (*P* = 0.026), mid anterolateral (*P* = 0.001), base inferior (*P* = 0.015), mid inferior (*P* = 0.034), mid anterior (*P* = 0.015), base anteroseptal (*P* = 0.005), and mid anteroseptal (*P* = 0.009) in AUCM and DCM. The segmental strain increased in both groups from the basal to the apex.

**Table 2:** Comparison of echocardiographic parameters in study population

Values	DCM group	AUCM group	*P* value
LVEDD^a^ (cm)	5.82 ± 0.92	5.42 ± 0.65	0.081
LVESD^b^ (cm)	4.45 ± 1.28	3.89 ± 0.67	0.066
LVEDV^c^ (mL)	116.09 ± 47.15	121.88 ± 36.63	0.631
LVESV^d^ (mL)	78.38 ± 44.73	78.23 ± 36.49	0.805
LV mass index (g/m^2^)	223.62 ± 78.37	251.52 ± 71.07	0.198
LA size (cm)	4.30 ± 0.63	3.93 ± 0.79	0.328
IVSD^e^ cm)	1.17 ± 0.08	1.10 ± 0.23	0.508
LVEF-2D (%)^f^	27.79 ± 13.52	29.58 ± 10.38	0.601
*e*′ Septal (Cm/s)	6.85 ± 2.79	6.19 ± 1.56	0.302
*E*/*e*′	13.86 ± 9.45	13.69 ± 5.39	0.942
*E*/*A*	1.20 ± 0.52	1.27 ± 0.87	0.769
*E* deceleration time (ms)	248.64 ± 77.68	176.00 ± 64.53	0.011*
Systolic PAP^g^ (mmHg)	34.52 ± 11.13	43.00 ± 11.99	0.015*
TAPSE^h^ (cm)	2.02 ± 0.42	1.92 ± 0.29	0.420
LV Tei index	0.58 ± 0.25	0.71 ± 0.31	0.111
LV S’(cm/s)	6.28 ± 2.04	5.88 ± 1.53	0.437
MAPSE^i^ (cm)	1.24 ± 0.06	1.25 ± 0.48	0.106
GLS^j^%	− 14.34 ± 4.8	− 11.98 ± 4.88	0.045*

*P value < 0.05

^a^Left ventricular end-diastolic diameter

^b^Left ventricular end-systolic diameter

^c^Left ventricular end-diastolic volume

^d^Left ventricular end-systolic volume

^e^Inter ventricular septum diameter

^f^Left ventricular Ejection Fraction

^g^Pulmonary Artery Pressure

^h^Tricuspid Annular Plane Systolic Excursion

^i^Mitral Annular Plane Systolic Excursion

^j^Global Longitudinal Strain

**Table 3:** Compared segmental strains in patients with advanced uremic cardiomyopathy and dilated cardiomyopathy

Segmental strains %	DCM group	AUCM group	*P* value	Segmental strains %	DCM group	AUCM group	*P* value
Inferoseptal wall				Inferior wall			
Apex	− 17.91 ± 6.49	− 17.04 ± 6.57	0.624	Apex	− 16.62 ± 6.09	− 16.64 ± 7.72	0.995
Mid	− 13.62 ± 6.35	− 10.40 ± 5.88	0.048	Mid	− 14.33 ± 6.09	− 10.99 ± 4.47	0.034
Base	− 10.45 ± 6.18	− 8.62 ± 5.6	0.259	Base	− 11.95 ± 5.79	− 7.77 ± 5.93	0.015
Anterolateral wall				Anterior wall			
Apex	− 16.79 ± 5.86	− 13.19 ± 8.86	0.099	Apex	− 16.50 ± 6.30	− 12.26 ± 10.25	0.088
Mid	− 13.83 ± 5.27	− 6.13 ± 9.29	0.001	Mid	− 14.00 ± 6.95	− 9.46 ± 7.25	0.015
Base	− 11.25 ± 7.25	− 5.68 ± 9.79	0.026	Base	− 11.29 ± 7.49	− 7.46 ± 8.59	0.099
Anteroseptal wall				Inferolateral wall			
Mid	− 16.50 ± 6.53	− 11.07 ± 7.51	0.009	Mid	− 14.12 ± 6.33	− 11.58 ± 7.75	0.212
Base	− 12.83 ± 6.77	− 6.88 ± 7.35	0.005	Base	− 11.04 ± 8.71	− 8.81 ± 8.72	0.252
Apical cap							
	− 17.33 ± 5.59	− 16.19 ± 6 .81	0.522

Also, those suffering from AUCM had significantly lower mitral E wave deceleration time (*P* = 0.011). Systolic PAP in AUCM was 43.00 ± 11.99 mmHg versus 34.52 ± 11.13 mmHg in the DCM group (*P* = 0.015).

No correlation was found between age and GLS (*P* value 0.18) and between RRT duration and GLS (*P* value 0.09).

## Discussion

Uremic cardiomyopathy is among the principal causes of morbidity and mortality in patients with ESRD. In recent years, comprehensive evaluations of cardiac involvement in ESRD patients have been performed. We assessed segmental longitudinal strain patterns in advanced uremic cardiomyopathy (AUCM) and compared this group of patients with DCM. The results showed that the global longitudinal strain was lower in AUCM versus DCM. Despite a lower strain in some myocardial segments in AUCM, both groups showed increased segmental longitudinal strain from base to apex. There was no specific segmental left ventricular strain pattern using speckle tracking to differentiate advanced uremic cardiomyopathy from DCM. GLS was reduced in advanced uremic cardiomyopathy and may indicate a more severe myocardial injury. The major limitation of this conclusion is the significant difference between the age of study participants in AUCM and DCM in our study; AUCM patients were one decade younger than DCM. The important question is the effect of aging on GLS values. In one meta-analysis, the effect of aging on GLS was assessed. The decrease in GLS was most pronounced after sixty in the normal population [12]. The lower values of GLS in younger ESRD patients with AUCM denote the greater degree of myocardial involvement in our study.

Various studies have evaluated and compared echocardiographic parameters between patients with uremic cardiomyopathy and healthy individuals. In the study by Tamulnait et al., diastolic dysfunction was reported in 81.6% of patients with ERSD. Also, the ESRD group's mean GLS and global circumferential strain (GCS) were significantly lower than healthy controls. RV GLS was significantly lower in ESRD patients than in healthy controls [13]. In a study by Hassanin et al. patients with CKD had lower LV GLS and RV GLS than healthy subjects [14]. Two other studies also showed that LV GLS, LV GCS, and LV global radial strain (GRS) in patients with ESRD were significantly lower than those who underwent kidney transplantation or the control group [15, 16]. The results of this presenting study emphasize that myocardial injury in patients with advanced uremic cardiomyopathy is more severe than DCM. Hence, early and appropriate treatment of uremia should be considered.

The study's main limitations were the small number of patients and the absence of long-term follow-up for comparing cardiovascular events and mortality. The advanced uremic and dilated cardiomyopathy were not matched properly regarding the age of the study population. The study did not include the duration of the severe decline of the eGFR to the initiation of the first hemodialysis session. Meanwhile, data on mean arterial noninvasive blood pressure measurement, renal function tests, mean eGFR, and hemoglobin levels in both groups needed to be documented.

## Conclusions

Myocardial injury in patients with advanced uremic cardiomyopathy is more severe than DCM. There is no specific pattern of segmental strain to differentiate uremic cardiomyopathy and DCM.

## Data Availability

The data of this study are available for further analysis upon request.

## Abbreviations

*ESRD*: End-stage renal disease
*AUCM*: Advanced uremic cardiomyopathy
DCM: Dilated cardiomyopathy
GLS: Global longitudinal strain
STE: Speckle tracking echocardiography
CKD: Chronic kidney disease
LVH: Left ventricular hypertrophy
AVF: Atriovenous fistula
LVEF: Left ventricular ejection fraction
TDI: Tissue doppler imaging
GCS: Global circumferential strain
GRS: Global radial strain
